# Effects of COVID-19 Infection on the Oral Glucose Tolerance Test Results in Pregnancy

**DOI:** 10.7759/cureus.46404

**Published:** 2023-10-03

**Authors:** Cevat Rifat Cundubey, Mehmet Ak, Bertan Demir, Seyma Cam

**Affiliations:** 1 Obstetrics and Gynecology, Kayseri City Training and Research Hospital, Kayseri, TUR

**Keywords:** neonatal outcome, oral glucose tolerance test, pregnancy, gestational diabetes, covid-19

## Abstract

Introduction: The aim of this study was to evaluate the results of a 75 g oral glucose tolerance test (OGTT) performed in the second trimester of pregnancy, the prevalence of gestational diabetes mellitus (GDM), and perinatal outcomes in pregnant women with a history of coronavirus disease 2019 (COVID-19) infection and to examine the effect of COVID-19 infection history on OGTT results and GDM prevalence.

Methods: We retrospectively analyzed the data of 463 patients who visited the Gynecology and Obstetrics Department of Kayseri City Hospital between March 2020 and January 2023 and were administered a 75-g OGTT in the second trimester of their pregnancy. Accordingly, we traced COVID-19 history, OGTT results, GDM prevalence, and newborn outcomes among the patients.

Results: OGTT glucose values were higher in the study group with a history of COVID-19 infection, but there was no significant difference between the groups. GDM developed in 13 (23.2%) pregnant women in the group with a history of COVID-19 infection and 88 (21.6%) pregnant women in the control group without a history of COVID-19 (p: 0.348). In addition, in pregnant women diagnosed with GDM, insulin requirement was 8.9% in the COVID-19 (+) group and 5.1% in the COVID-19 (-) group, and the results were not statistically significant (p: 0.178). There was no significant difference in neonatal outcomes between the groups.

Conclusions: In our study, we found that glucose values were higher and the prevalence of GDM was higher in pregnant women with a history of COVID-19 infection before the OGTT. It is necessary to be more careful about issues such as blood glucose regulation and GDM risk in pregnancy follow-up after infections such as COVID-19, which may have widespread systemic inflammatory effects, and patients should be informed in detail for pregnancy follow-up.

## Introduction

Coronaviruses are renowned for being prominent human and animal pathogens. Exponentially spreading pneumonia cases in Wuhan, China toward the end of 2019 were discovered to be caused by a novel coronavirus. The spread of the virus was unstoppable, causing an outbreak across China, followed by an unprecedented number of pneumonia cases worldwide. In February 2020, the World Health Organization (WHO) identified the cases as coronavirus disease 2019 (COVID-19) caused by severe acute respiratory syndrome coronavirus 2 (SARS-CoV-2) [1].

The virus can be transmitted by droplets from infected individuals through coughing, sneezing, and contact with contaminated surfaces and the patient's hand to the mucous membranes of the mouth, eyes, and nose [2]. Transmission from asymptomatic individuals has also been reported [3]. Common symptoms of infection include respiratory symptoms of cough, fever, and dyspnea. In more severe cases, pneumonia, severe acute respiratory infection, renal failure, and even death can develop.

COVID-19 appears to precipitate severe diabetes symptoms, including diabetic ketoacidosis, hyperosmolar hyperglycemic state, and severe insulin resistance [4]. The previous research demonstrated that COVID-19 can enter pancreatic islet cells through angiotensin-converting enzyme-2 (ACE-2) receptors and cause reversible β-cell damage and transient hyperglycemia through this mechanism, the possibilities for potential new-onset diabetes triggered by COVID-19 have increased [5]. Similarly, the relevant literature emphasized that SARS-CoV-2 may damage pancreatic beta cells, which may lead to an increase in the incidence of newly diagnosed diabetes mellitus (DM) [6,7].

A follow-up study investigating the pathogenesis of pancreatic lesions in 2010 found that pancreatic islets were strongly immune-positive for ACE2, while exocrine tissues were only weakly positive. DM developed in 20 of 39 patients who did not receive corticosteroids during SARS illness, and these patients required early insulin treatment, possibly due to the destruction of pancreatic beta cells or increased insulin resistance due to multiple mechanisms. Six of them were diagnosed with DM while being discharged [8]. It should be noted that new-onset cases of hyperglycemia are increasingly being identified with COVID-19 in adults without a prior history of diabetes. While infection-induced inflammation and cytokine activation and resulting insulin resistance may lead to stress hyperglycemia, it is not yet known to what extent direct viral destruction of islet cells with reduced insulin production and secretion may contribute to it [9].

Glucose intolerance resulting in hyperglycemia that is first diagnosed during pregnancy is often called gestational diabetes mellitus (GDM) [10-12], and the gold standard screening for GDM is known to be with a 75-g oral glucose tolerance test (OGTT) between 24 and 28 weeks of pregnancy. The result is always considered positive when a single OGTT value is found to exceed the respective threshold value [13]. GDM is one of the most common pregnancy complications with a global prevalence of 13.4% and a European prevalence of 7.8% [14,15].

Pregnancy is a process that makes women vulnerable to viral infections and causes partial suppression of the immune system. Even in seasonal influenza seen especially in winter months, morbidity rates increase during pregnancy. There are studies showing that COVID-19 infection during pregnancy is risky [16,17]. In the literature, studies showed that in case of viral transmission, maternal comorbidities such as pre-pregnancy and/or gestational diabetes, adverse pregnancy outcomes, and severe course of COVID-19 infection constitute a particularly high risk [18]. In reviews and meta-analyses of several studies, diabetes has been associated with a more severe course of COVID-19 and increased mortality [19,20]. It has also been shown that newborns born to women with COVID-19 are at high risk of morbidity and mortality [21]. In addition, the new onset of diabetes has been described in the course of COVID-19 infection [22]. This suggests a bidirectional relationship between COVID-19 and diabetes [23,24].

In our literature review, we found that there are very few studies on the possible relationship between COVID-19 and GDM. The aim of our study was to evaluate the results of the 75 g OGTT performed in the second trimester of pregnancy, GDM prevalence, and perinatal outcomes in pregnant women with a history of COVID-19 infection and to examine the effect of COVID-19 infection history on OGTT results and GDM prevalence.

## Materials and methods

We carried out this study with the ethical approval of the Ethics Committee of Kayseri City Hospital (No.: 393 dated 04.29.2021) and in accordance with the principles in the Declaration of Helsinki. In this retrospective, single-center study, we considered the data of patients who visited the Gynecology and Obstetrics Department of Kayseri City Hospital between March 2020 and January 2023 and were administered a 75-g OGTT in the second trimester of their pregnancy. While the COVID-19 positive (+) group consisted of pregnant women who had a positive result of nasopharyngeal swab and antibody tests before the OGTT, those without a history of COVID-19 infection and with negative test results were included in the control group. We then noted down their demographic characteristics, OGTT results, GDM status, mode of delivery, pre-pregnancy body mass index (BMI), and newborn outcomes.

Nevertheless, we excluded those with pre-pregnancy diabetes (type 1, type 2, or others), pre-pregnancy endocrine disorder (Cushing’s syndrome, Addison’s disease), a history of bariatric surgery, malabsorption diseases (Crohn’s disease, ulcerative colitis, celiac disease), and multiple pregnancies. We also excluded those not receiving the diagnostic OGTT within the recommended time interval (24+0 to 27+6 weeks of gestation) or prior to the COVID-19 infection. Therefore, we analyzed the data of 463 pregnant women, 56 in the patient group and 407 in the control group.

GDM is typically diagnosed based on the 75-g OGTT results, using the criteria suggested by the International Association of Diabetes and Pregnancy Working Groups (IADPSG). These criteria involve glucose levels exceeding the following thresholds: 92 mg/dL when fasting, 180 mg/dL during the first hour, and 153 mg/dL during the second hour (10). Fasting glucose exceeding 126 mg/dL or glucose values exceeding 200 mg/dL twice are considered pre-existing diabetes [25,26].

Statistical analysis

Statistical analysis was performed using IBM SPSS Statistics for Windows, Version 20 (Released 2011; IBM Corp., Armonk, New York, United States). Data are presented as median or number (percentage) with 95% confidence interval (CI). In addition, chi-square tests were performed for categorical variables and t-tests were performed to compare the means of two groups. All analyses were performed using a two-sided model, taking into account normal distribution as appropriate. Multiple logistic regression was performed to identify factors associated with the incidence of the primary outcome of interest. A p-value less than 0.05 was considered statistically significant.

## Results

In our study, 463 pregnant women who underwent 75 g OGTT screening between 24 and 28 weeks of pregnancy between 03/2020 and 03/2022 in our clinic were included. It was determined that 56 of the pregnant women (12%) had COVID-19 infection before the OGTT test, while the remaining 407 pregnant women (88%) did not have COVID-19 in their history and tests performed. Demographic characteristics and neonatal outcomes of both groups are presented in Table 1.

**Table 1:** Demographic characteristics and neonatal outcomes of pregnant women with and without a history of COVID-19 BMI: Body mass index; CI: confidence interval; NICU: neonatal intensive care unit; COVID-19: coronavirus disease 2019

	COVID-19 (+) (n = 56)	COVID-19 (-) (n = 407)	p-value
Maternal age, mean, (%95 CI), year	31.2 ± 2.8	30.6 ± 3.1	0.417
Parity, mean, (%95 CI)	1.3 ± 0.9	1.2 ± 1.1	0.289
BMI, mean, (%95 CI), (kg/m^2^)	28.6 ± 2.9	27.4 ± 2.4	0.253
Week of delivery, median (IQR), hafta	39.3 (38,5-40.1)	39.2 (38.1-40.3)	0.188
Cesarean section rate (n, %)	25 (44.6)	167 (41.0)	0.412
Apgar 1-min, mean, (%95 CI)	8 (8-9)	8 (8-9)	0,514
Apgar 5-min, mean, (%95 CI)	9 (8-10)	9 (8-10)	0,611
Fetal birth weight, mean, (%95 CI), gr	3472 (2891-3946)	3381 (2934-3898)	0,225
Meconium ratio (n, %)	2 (3,5)	12 (2,9)	0,114
Nicu admission (n, %)	1 (1,7)	5 (1,2)	0,164

Fasting glucose, glucose at 60 minutes, and glucose at 120 minutes were higher in the COVID-19 (+) group than in the COVID-19 (-) group, but no statistically significant difference was observed between the groups (p: 0.122, p: 0.166, p: 0.236). OGTT results of the groups are presented in Table 2. 

**Table 2:** Analysis of the BMI factor that may increase the frequency of GDM diagnosis BMI: Body mass index; CI: confidence interval; HR: hazard ratio; GDM: gestational diabetes mellitus

	COVID (+) HR (%95 CI)	p-value	COVID (-) HR (%95 CI)	p-value
VKİ > 25 kg/m^2^	1,48 (1,06-1,72)	0,086	1,62 (1,04-1,98)	0,079

GDM developed in 13 (23.2%) pregnant women in the COVID-19 (+) group and 88 (21.6%) pregnant women in the COVID-19 (-) group, and there was no significant difference in the rate of GDM between the groups (p: 0.348). In addition, in pregnant women diagnosed with GDM, insulin requirement was 8.9% in the COVID-19 (+) group and 5.1% in the COVID-19 (-) group, and the results were not statistically significant (p: 0.178). GDM and insulin requirement rates of the groups are presented in Table 3. 

**Table 3:** Comparison of OGTT values in pregnant women with and without a history of COVID-19 OGTT: 75-g oral glucose tolerance test; CI: confidence interval; COVID-19: coronavirus disease 2019

OGTT values,mean(%95 CI), mg/dl	COVID-19 (+) (n = 56)	COVID-19 (-) (n = 407)	p-value
Fasting glucose (mg/dL)	84.3 ± 7.6	81.2 ± 8.9	0.122
OGTT 60-min. (mg/dL)	160.7 ± 21.2	152.6 ± 18.3	0.166
OGTT 120-min. (mg/dL)	120.6 ± 15.4	112.3 ± 13.6	0.236

The effect of BMI on the frequency of GDM diagnosis is presented in Table 4. Although the rate of GDM is similar in pregnant women with a history of COVID-19, it has been found to be higher than those who do not need insulin.

**Table 4:** Comparison of GDM and insulin requirement rates in pregnant women with and without a history of COVID-19 GDM: Gestational diabetes mellitus; COVID-19: coronavirus disease 2019

	COVID (+)(n:56)	COVID(-)(n:407)	p-value
GDM (n,%)	13 (23.2)	88 (21.6)	0.348
Insulin requirement (n,%)	5 (8.9)	21 (5.1)	0.178

## Discussion

The literature hosts a plethora of studies addressing the link between COVID-19 infection and type 2 diabetes [20,21]. Nevertheless, possible associations between COVID-19 infection and GDM or blood sugar imbalance during pregnancy have been explored insufficiently. Even there is a paucity of research comparing OGTT results of pregnant women with and without a history of COVID-19. Our findings showed comparable glucose levels and an increased GDM prevalence as a result of the diagnostic OGTT in 24-28 weeks of pregnancy among our patients.

Although the exact pathophysiological bidirectional relationship of SARS-CoV-2 infection with diabetes has not yet been fully understood, previous research reported an increased incidence of new-onset diabetes during the COVID-19 disease [22,23]. It is hypothesized that the virus binds to the ACE-2 receptor, allowing the virus to enter cells. The ACE-2 receptor is expressed on the surface of several major metabolic organs, particularly on pancreatic beta cells. In the case of viral infection, beta cell dysfunction may occur, which may explain the deterioration in metabolic status observed in DM(+) infected patients [23]. Overlapping the previous findings, we discovered that the COVID (+) group had higher insulin requirement and GDM prevalence than the control group.

COVID-19 or other viral infections (e.g., hepatitis C) may impair beta cell function, leading to type 2 diabetes and even triggering the onset of type 1 diabetes [24,25]. Thus, viral infections should be minded among pregnant women whose insulin sensitivity is already physiologically reduced [26]. In this regard, the high GDM prevalence in our COVID (+) group may be considered consistent with the existing literature.

The incidence of GDM was found to be higher in women with COVID-19 infection [27]. Similar research also documented an increase in the incidence of GDM globally during the pandemic due to lifestyle changes [28-31]. Similar to the existing literature, we discovered GDM prevalence to be higher among pregnant women with a history of COVID-19. In addition, GDM prevalence was found to be higher than reference values both in the patient and control groups (42.3% vs. 23.8%), which also overlaps with the previous results. This picture may have been led by complete closures and lifestyle changes among pregnant women during the pandemic.

## Conclusions

In our study, we found that glucose values were higher and the prevalence of GDM was higher in pregnant women with a history of COVID-19 infection before the OGTT. We concluded that COVID-19 infection may cause glucose intolerance in pregnancy, so caution should be exercised. While the need for insulin is normally limited in women with GDM, it should be kept in mind that the need for insulin increases with a history of COVID-19, and dysglycemia treatment in GDM patients with a history of COVID-19 may require enhanced care in terms of blood glucose regulation.
